# The Homœopathic Medical Society of Ohio

**Published:** 1882-05

**Authors:** H. E. Beebe

**Affiliations:** Secretary; Sidney, Ohio


### THE HOMŒOPATHIC MEDICAL SOCIETY OF OHIO.

SIDNEY, OHIO, April 1st, 1882.

DEAR DOCTOR: The Eighteenth Annual Session of the Homœopathic Medical Society of Ohio, will be held in the city of Springfield, May 9th and 10th. The interest manifested in this meeting is more than ordinary; a large attendance is anticipated. These flattering prospects should induce each homœopathic physician in the State to strain every point to be present. We owe it to our patrons, to the cause and to ourselves.

Fraternally,

H. E. BEEBE, *Secretary*.

